# Targeting and functional effects of biomaterials-based nanoagents for acute pancreatitis treatment

**DOI:** 10.3389/fbioe.2022.1122619

**Published:** 2023-01-10

**Authors:** Yujie Cai, Qian Cao, Jiannan Li, Tongjun Liu

**Affiliations:** ^1^ Department of Colorectal and Anal Surgery, The Second Hospital of Jilin University, Changchun, China; ^2^ Department of Education, The Second Hospital of Jilin University, Changchun, China

**Keywords:** acute pancreatitis, passive targeting, active targeting, nanotechnology, biomaterials

## Abstract

Acute pancreatitis (AP) is a severe life-threatening inflammatory disease showing primary characteristics of excessive inflammatory response and oxidative stress. Based on the pathophysiology of AP, several anti-inflammatory and anti-oxidative stress agents have been studied. However, the low accumulated concentrations and scattered biodistributions limit the application of these agents. With the development of nanotechnology, functional nanomaterials can improve the bioavailability of drugs and extend their half-life by reducing immunogenicity to achieve targeted drug delivery. The biomaterial-based carriers can mediate the passive or active delivery of drugs to the target site for improved therapeutic effects, such as anti-oxidation and anti-inflammation for AP treatment. Other biomaterials-based nanomedicine may exhibit different functions with/without targeting effects. In this review, we have summarized the targeting and functional effects of biomaterials-based nanoagents specifically for AP treatment.

## 1 Introduction

Acute pancreatitis (AP) is an unpredictable and potentially fatal inflammatory gastrointestinal disease. The global incidence of AP ranges from 13 to 45 cases per 100,000 people and is increasing annually (Petrov and Yadav, 2019). Studies have shown that alcoholism, smoking, biliary tract abnormalities, and autoimmune diseases usually cause AP, which can be accompanied by epigastric pain, nausea, vomiting, bloating, systemic involvement, and organ failure (Lankisch et al., 2015). According to the clinical severity, AP can be divided into mild, moderate, and severe categories (Ratia Gimenez et al., 2014). Mostly, mild AP can be cured automatically after progressing to a certain extent (Lankisch et al., 2015). Approximately 20% of patients with pancreatitis develop moderate to severe AP, which is difficult to cure, and show symptoms of pancreatic necrosis and/or organ failure accompanied by long-term recurrent episodes in most patients with irreversible pathological changes (Lee and Papachristou, 2019; Mederos et al., 2021). AP can cause exocrine dysfunction of the pancreas and damage acinar cells. Some severe AP cases can progress to systemic inflammatory responses, multiple organ damage, or function failure, with a mortality rate of as high as 20% (Mederos et al., 2021).

Oxygen free radicals (OFRs) and derivatives play an important role in AP progression and pancreatic tissue damage; hydrogen peroxide, superoxide anion, hydroxyl group, and singlet oxygen are the main factors causing cell damage (Padureanu et al., 2022). OFRs can promote the adhesion, activation, and migration of leukocytes, damage the integrity of endothelial cells, and increase the permeability of capillaries, thus leading to the loss of circulating blood volume, microcirculation disorders, and aggravated pancreatic injury (Padureanu et al., 2022). During the AP-mediated inflammatory response process, inflammatory factors and oxidative stress play a synergistic role *via* triggering a common signaling pathway, mainly activated by MAPK and NF-κB, leading to an amplification of inflammation cascade (Zhang et al., 2022a). Based on the pathophysiology of AP, various anti-oxidative and anti-inflammatory drugs have been explored (Zhang et al., 2022b). However, because of the limited solubility, short half-life, and poor stability, these traditional drugs cannot pass through the blood-pancreas barrier to reach sufficient concentration at the inflammation site (Zhou et al., 2019). Hence, a safe and effective treatment method is yet to be developed to achieve improved anti-oxidation and anti-inflammation effects for AP treatment in the clinic.

In recent years, nanotechnology has been widely applied in the biomedicine field, with rapid progress in the prevention, diagnosis, and treatment of diseases (Gagliardi et al., 2021; Wang et al., 2021). Nanoparticles of different shapes, pore sizes, and structures can be utilized in the development of nanoscale drug delivery systems using polymeric and inorganic materials as carriers (Atiyah et al., 2022). Nanomedicines can improve drug efficacy, safety, physicochemical properties, and pharmacokinetics or pharmacodynamics (Atiyah et al., 2022). Additionally, functional nanomaterials can enhance the bioavailability of oral drugs *in vivo* and extend their half-life by reducing immunogenicity to achieve targeted drug delivery (Hou et al., 2021; Mallakpour et al., 2022). For AP treatment, biomaterials can act as nanocarriers or nanomedicines for passive or active delivery of drugs to the target site, which is conducive to drug accumulation at the inflammatory pancreatic area, for improved therapeutic effects, including anti-oxidation and anti-inflammation (Zhang et al., 2022b). Other biomaterials-based nanomedicine may possess different functions with/without targeting effects for AP treatment. In this review, we have summarized and discussed the targeting and functional effects of biomaterials-based nanoagents for AP treatment.

## 2 Role of biomaterials-based nanoagents in AP treatment

**FIGURE 1:**
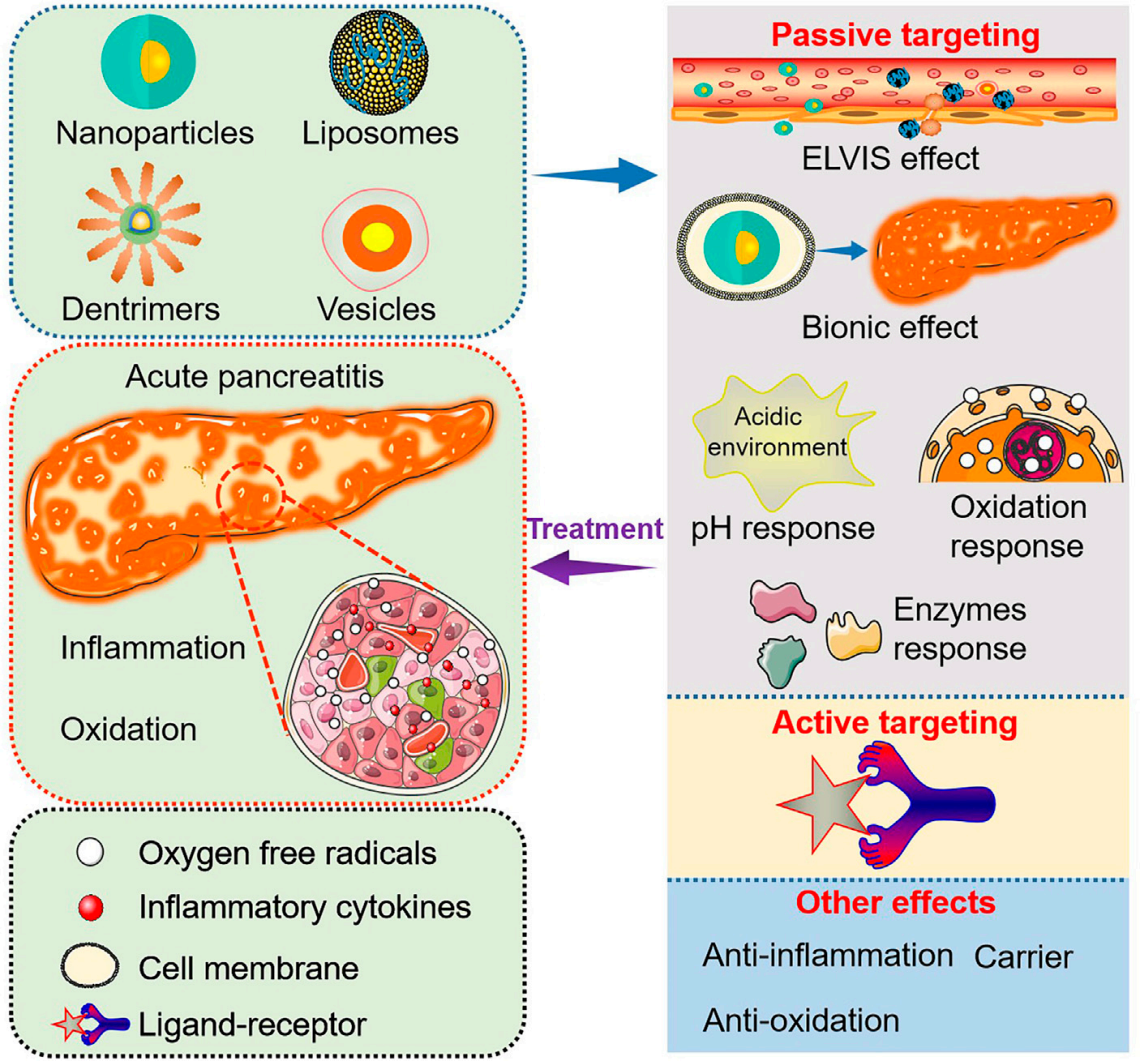
Targeting and functional effects of biomaterials-based nanoagents constructed specifically for AP treatment.

Although several important roles of biomaterials as drug carriers and nanomedicine for disease diagnosis and treatment have already been discussed (Festas et al., 2020; Gagliardi et al., 2021), this review mainly outlines the targeting and functional effects of biomaterials-based nanoagents constructed specifically for AP treatment (Figure 1; Table 1).

**TABLE 1:** The targeting and functional effects of biomaterials constructed for nanoagents for AP treatment.

Targeting category	Materials	Formulation	Loaded agents	Purpose of constructed biomaterials	Delivery route	Model	Main results	Ref
Passive (ELVIS effect, enzyme response)	Silk fibrin	Nanoparticles	Bilirubin	Carrier	Intravenous injection	L-Arginine induced rat model	Bilirubin-loaded silk fibrin nanoparticles prevented NF-kappa B pathway and activated the Nrf2/HO-1 pathway to inhibit oxidative stress and inflammatory responses	Yao et al. (2020)
Passive (ELVIS effect)	The nanoliposomes were prepared by thin layer evaporation technique, and L-α-phosphatidylcholine and cholesterol were mainly used	Liposomes	Caffeic acid phenethyl ester (CAPE)	Carrier	Oral delivery	L-ornithine induced rat model	The CAPE-loaded nanoliposomes decreased the pancreatic secretions, oxidative stress, local inflammation, tissue apoptosis, and impaired energy status for the treatment of pancreatitis	Shahin et al. (2022)
Passive (ELVIS effect)	PLGA	Nanoparticles	Curcumin	Carrier	Intravenous injection	Cerulein induced rat model	Cur-loaded nanoparticles significantly decreased serum amylase and lipase levels, oxidative and nitrosative stress, and the expression of inflammatory cytokines	Anchi et al. (2018)
Passive (ELVIS effect)	Rebaudioside A (RA)	Micelles	Empagliflozin (EMP)	Carrier	Oral delivery	L-Arginine induced rat model	The RA-EMP micelles performed the therapeutic effects towards AP by suppressing oxidative stress and proinflammatory cytokines	Li et al. (2022)
Passive (ELVIS effect, Bionic targeting)	PEG-PLGA coated with neutrophil membranes	Nanoparticles	Celastrol	Carrier, to drive to the inflammation site *via* chemokine recruitment	Intravenous injection	Sodium taurocholate induced rat model	The composite reduced serum amylase levels, pro-inflammatory cytokines, and inhibited systematic side effects	Zhou et al. (2019)
Passive (ELVIS effect, pH response)	Silica	Nanoparticles	Chitosan oligosaccharides (COSs)	Carrier	Intraperitoneally injection	Cerulein induced rat model	The COSs-loaded silica nanoparticles can activate Nrf2 and suppress NF-κB and the NLRP3 inflammasome for ameliorating AP.	Mei et al. (2020)
Passive (oxidant response)	Yttrium oxide (Y_2_O_3_)	Nanoparticles	_	Performing direct antioxidant activity	Intraperitoneally injection	Cerulein induced rat model	The nanocomposite decreased oxidative stress and attenuated the mitochondrial stress and inflammatory markers	Khurana et al. (2019)
Passive (ELVIS effect, Bionic targeting, enzyme response)	Neutrophil membrane-coated silk fibroin (SF)-nanoparticles	Nanoparticles	Ferulic acid (FA)	Carrier	Intravenous injection	Not mentioned	The nanoparticles can targeted deliver FA to inflammatory pancreas lesion and perform anti-inflammation and anti-oxidation effects	Hassanzadeh et al. (2021)
Passive (Bionic targeting, enzyme response)	PLGA nanoparticles coated with macrophage (MΦ) membrane modified with melittin and MJ-33	Nanoparticles	_	Lure and kill PLA2 enzymes	Intravenous injection	Cerulein induced rat model	These nanoparticles can suppress PLA2 activity and preventing inflammatory responses, therefore decreasing tissue damage in pancreas	Zhang et al. (2021)
Active targeting	Peptide-conjugated pegylated DOPC liposomes	Liposomes	Apigenin	Carrier, active target through specific peptide	Intravenous injection	Cerulein induced rat model	Increasing the apigenin accumulation in pancreas for performing acini preservation and reducing oxidative stress	Hung et al. (2021)
_	Prussian blue nanozymes were prepared by polyvinylpyrrolidone modification method	Nanoparticles	_	To drive intrinsic ROS scavenging and inflammation inhibiting properties	Intravenous injection	Cerulein induced rat model	Prussian blue nanozymes inhibited toll-like receptors (TLRs)/NF-κB signaling pathway, thus decreasing the inflammation responses and oxidative stress for AP treatment	Zhang et al. (2022c)
_	Generation 5 (G5) polyamidoamine (PAMAM) dendrimers with two different surface groups, G4.5-COOH and G5-OH	Dendrimers	_	Performing anti-inflammatory effects	Intravenous injection	Cerulein induced rat model	G4.5-COOH and G5-OH inhibited decreased the expression of pro-inflammatory cytokines by suppressing nuclear translocation of NF-κB in macrophages	Tang et al. (2015)
_	Tetrahedral framework nucleic acids	Nanoparticles	_	Suppressing inflammation and preventing pathological cell death	Intravenous injection	Taurocholate induced rat model	Inhibiting inflammatory cytokines in tissues and blood	Wang et al. (2022)
_	Carbon monoxide bound hemoglobin vesicles (CO-HbV)	Vesicles	_	Acting as a donor for CO and oxygen carrier after releasing CO.	Intravenous injection	Choline-deficient ethionine-supplemented diet induced rat model	CO-HbV decreased pro-inflammatory cytokines expression, neutrophil infiltration, oxidative injuries in pancreatic tissue, and systematic side effects	Nagao et al. (2016)
_	Lipid-based liquid crystalline nanoparticles with the lipid mixture of phosphatidylcholine (PC), glycerol dioleate (GDO) and polysorbate 80 (P80)	Nanoparticles	Somatostatin	Carrier	_	_	Extending plasma half-lives of somatostatin	Cervin et al. (2009)

### 2.1 Targeting effects

Targeting effect is crucial for the treatment of various diseases and can be divided into three aspects, i.e., passive, active, and physical targeting (Russ and Wagner, 2007; Ivey et al., 2016; Kanamala et al., 2016). Accumulation of nanoagents at the target sites for enhanced therapeutic efficacy while reducing systematic side effects can be achieved through targeting effect (Steichen et al., 2013). According to the existing literature, biomaterials-based targeting effects for AP treatment are mainly passive and active (Zhang et al., 2022b).

#### 2.1.1 Passive targeting

During passive targeting, biomaterials-based nanocarriers generally act via physical and chemical interactions (hydrophobic and electrostatic interactions) as well as physical factors (such as carrier size and mass) to achieve targeted drug delivery (Attia et al., 2019). For pancreatitis, passive targeting can be achieved through increased permeability of tissue space and other microenvironment biochemical properties, including pH, reactive oxygen species (ROS), and digestive enzymes (Chiorean and Coveler, 2015).

Inflammatory responses can increase the permeability of blood vessels, subsequently triggering the extravasation through leaky vasculature and inflammatory cell-mediated sequestration (ELVIS) (Gong et al., 2019), which allows passive targeting of suitable size range nanoparticles for accumulation at the inflammation sites (Ren et al., 2019). Yao et al. (2020) developed silk-fibrin (SF)-based nanoparticles encapsulating bilirubin, the main anti-oxidant component of heme catabolism, for AP treatment. The animal experiment showed that the synthesized nanoparticles could selectively target the inflammatory sites in the pancreas to release bilirubin. The bilirubin-loaded SF nanoparticles prevented the NF-κB pathway and activated the Nrf2/HO-1 pathway to inhibit oxidative stress and inflammatory responses. In another study, L-α-phosphatidylcholine and cholesterol were mainly used to prepare nanoliposomes by thin layer evaporation technique (Shahin et al., 2022), loaded with caffeic acid phenethyl ester (CAPE) for oral administration in a rat model. The CAPE-loaded nanoliposomes decreased pancreatic secretions, oxidative stress, local inflammation, tissue apoptosis, and impaired energy status as therapeutic effects for AP treatment. The *in vivo* biodistribution study of the nanoliposomes was not performed; thus, the targeting effects could not be supported directly. However, the enhanced treatment efficacy was attributed to the altering tissue biodistribution of smaller-size liposomes and cellular uptake mechanisms (Shahin et al., 2022). Notably, liposomes particle size is their most influential characteristic affecting the circulation time and biodistribution after intravenous injection, which plays a vital role in passing through the leaky vasculature and accumulation at inflammatory sites *in vivo* (Ren et al., 2019). Anchi et al. (2018) synthesized curcumin-loaded poly (lactic-co-glycolic acid) (PLGA) nanoparticles and evaluated the treatment efficacy in a cerulein induced AP model. The nanoparticles delivered curcumin to the inflammatory pancreatic site mediated by the ELVIS effect, resulting in a significant reduction of serum amylase and lipase levels, oxidative and nitrosative stress, and the expression of inflammatory cytokines. In addition, other previous studies have also reported the passive targeting of biomaterials-based nanocarriers *via* the ELVIS effect for AP therapy. Li et al. (2022) reported empagliflozin (EMP) loaded rebaudioside A (RA) micelles with a particle size of 5.234 ± 0.311 nm. The small particle size of the synthesized RA-EMP micelles (less than 20 nm) could benefit their cellular uptake and tissue accumulation for therapeutic effects against AP in a rat model by suppressing oxidative stress and proinflammatory cytokines.

Biomaterials-based nanoparticles with small size triggering the ELVIS effect at inflammatory sites have been applied in drug delivery and treatment of various inflammatory diseases. However, traditional nanoparticles are easily recognized by the reticuloendothelial system *in vivo* and are difficult to accumulate passively at the inflammation area via the ELVIS effect. For example, although the small particle size of RA-EMP micelles aided the passive targeting of EMP at the pancreatic inflammation site, the biodistribution study showed the highest EMP concentrations in kidney and liver tissues with reduced EMP accumulation in the pancreas, which could restrict the therapeutic efficacy (Li et al., 2022). Natural cell-membrane-modified nanoparticles have immense advantages in disease diagnosis and treatment due to the unique proteins, peptides, and enzymes present on the surface of cell membranes (Hwang et al., 2015) and are highly biocompatible to achieve extended circulation ability and/or target effects. Additionally, nanoparticles modified by partial or complete cell membranes can acquire the cell-derived bioactive property and homing effect for targeted drug delivery. Till now, membranes of various cell types, including red blood cells (Hu et al., 2011), platelets (Hu et al., 2015), white blood cells (Parodi et al., 2013), cancer cells (Chen et al., 2016), stem cells (Bose et al., 2018), etc., have been applied to modify nanoparticles for disease diagnosis and treatment. Utilizing the natural homing effect of cell membranes, nanoparticles can target the corresponding lesions to increase the therapeutic efficacy; the targeted homing effect is termed Bionic targeting (Liu et al., 2023). AP is an acute inflammatory disease characterized by the infiltration of a large number of inflammatory cells, including neutrophils and macrophages (Mayerle et al., 2012). Neutrophils are the most abundant type of granulocytes, accounting for 40 to 70 percent of all human white blood cells and the host’s first line of defense against invading pathogens and infections. The inherently phagocytic neutrophils can be activated by cytokines which then arrive at the inflammation sites (Selders et al., 2017; Castanheira and Kubes, 2019). Zhou et al. (2019) studied the treatment efficacy and mechanism of celastrol-loaded poly (ethylene glycol) methyl ether-block-PLGA (PEG-PLGA) nanoparticles coated with neutrophil membrane towards AP. The neutrophil membrane-modified nanoparticles aimed to endorse the Bionic targeting effect at the inflammation site through cytokines recruitment. In addition, the size of the applied nanoparticles (156.8 ± 2.3 nm) attributed to the passive ELVIS targeting effect. Furthermore, the modified celastrol-loaded nanoparticles reduced serum amylase levels and pro-inflammatory cytokines and inhibited systematic side effects in a sodium taurocholate-induced AP rat model.

The pH-responsive materials are usually capable of physical or chemical changes within a specific pH range (Lu et al., 2014). The pH-sensitive nanomaterials can achieve responsive release of drugs at the inflammatory site and correspondingly increase the drug accumulation (Kellum et al., 2004). Enhanced cellular metabolic activity leads to anaerobic glycolysis and lactic acid formation at the inflammation site (Zhang et al., 2020), resulting in an acidic microenvironment within the damaged pancreatic tissue. Applying pH-sensitive biomaterials to achieve passive targeting at the pancreatic tissues may further enhance the anti-oxidative and anti-inflammatory therapeutic effects. Silica nanoparticles, possessing porous structures and good biocompatibility, are usually used as carriers that can release drugs mediated by pH control to obtain their high concentrations within targeted tissues (Li et al., 2017; Zhang et al., 2018). Mei et al. (2020) developed silica-based nanoparticles encapsulating chitosan oligosaccharides (COSs) for their (COSs) targeted delivery to the pancreas, exerting anti-oxidation and anti-inflammation effects by activating Nrf2 and suppressing NF-κB and NLRP3 inflammasome for ameliorating AP. Moreover, the small size of the nanoparticles (305 nm) could also contribute to the ELVIS targeting effect.

The normal physiological ROS levels play an essential role in cell differentiation, proliferation, and migration (Hajam et al., 2022). In AP conditions, ROS are excessively produced in activated neutrophil and macrophage organelles such as mitochondria and endoplasmic reticulum. Overproduction of ROS can promote the production of inflammatory cytokines such as tumor necrosis factor alpha (TNFα), interleukin (IL) 1β, and IL 6, accelerating the progression of AP (Carrasco et al., 2014). The nanocomposites sensitive to oxidative response can be utilized in targeted therapy for AP. For example, intraperitoneal administration of yttrium oxide (Y_2_O_3_) nanoparticles decreased oxidative stress and attenuated the mitochondrial stress and inflammatory markers in an AP rat model (Khurana et al., 2019). The Y_2_O_3_ nanoparticles, like catalase and superoxide dismutase mimetic, possess promising anti-oxidation properties (Heckert et al., 2008).

During the trigger and development of AP, a variety of enzymes can be secreted by pancreatic tissues, such as proteolytic enzymes (Hu et al., 2020) and phospholipase A2 (PLA2) (Friess et al., 2001). Biomaterials-based nanoagents with enzyme response properties can achieve passive targeting effects at inflammatory sites to improve therapeutic mechanisms for AP. As discussed above, the SF protein in bilirubin-loaded SF nanoparticles, which induced the ELVIS passive targeting effects towards inflammatory pancreatic tissues (Yao et al., 2020), can be degraded by proteolytic enzymes, making SF a candidate material for enzyme-responsive nanoagents design (Zhao et al., 2015). As a result, the bilirubin-loaded SF nanoparticles also exhibited enzyme-responsive targeting effects for enhanced inhibition of oxidative stress and inflammatory responses (Yao et al., 2020). In another study, neutrophil membrane-coated SF-nanoparticles encapsulated with ferulic acid (FA) were developed for *in vivo* targeted delivery of FA to inflammatory pancreas lesions showing anti-inflammation and anti-oxidation effects (Hassanzadeh et al., 2021). The major components of the nanoparticles, SF and the entrapped neutrophil membrane, endowed the nanoagent with enzyme-responsive and Bionic targeting effects, respectively. Additionally, FA is a phenolic compound that has been applied in the treatment of various diseases, especially those accompanied by severe oxidative stress and inflammation responses (Zdunska et al., 2018). Zhang et al. (2021) synthesized macrophage (MΦ) membrane-coated PLGA nanoparticles modified with melittin and MJ-33 for AP treatment. Melittin is a short peptide with a high affinity for PLA2, while MJ-33 serves as a PLA2 inhibitor. The MΦ membrane and melittin in the nanocomposite may benefit the Bionic and enzyme-responsive targeting effects, respectively. In a rat AP model, these nanoparticles suppressed PLA2 activity, thus, preventing inflammatory responses and decreasing pancreatic tissue damage.

#### 2.1.2 Active targeting

Passive targeting based on the ELVIS effect and other mechanisms is insufficient due to the non-specific reaction between cells and nanoagents, leading to a significant reduction in cellular endocytosis of nanoagents. This affects the bioavailability of drugs and their therapeutic effects, resulting in drug leakage and resistance in the organism (Lee et al., 2022). To overcome these limitations, biomaterials-based agents with active targeting properties should be considered; specific ligands or non-serum based-biomarkers can be applied to increase the treatment efficacy of AP. The nanoparticles modified with specific peptides can recognize and bind with corresponding receptors expressed in certain cells in the inflammatory sites, resulting in enhanced drug accumulation (Da Silva-Candal et al., 2019; Lin et al., 2021). Hung et al. (2021) selected five pancreatitis-specific peptides using a computational-guided *in vivo* phage display approach, demonstrating selectivity to different pancreatic cells. The peptide-conjugated liposomes were encapsulated with apigenin for the targeted delivery of the drug to inflammatory pancreatic tissues for acinar cell preservation and oxidative stress reduction. Despite the advantages, the active targeting property of biomaterials-based nanoagents has not been studied and exploited extensively for AP treatment, highlighting the need for further research in this area.

### 2.2 Other functional effects

Besides the passive and active targeting effects, the biomaterials used for nanoagents construction may possess several other functional properties for AP treatment. Zhang et al. (2022b) developed polyvinylpyrrolidone (PVP)-modified molybdenum selenide two-dimensional nanosheets (MoSe_2_@PVP NSs), which could act as artificial enzymes and mimic multi-enzyme activities, including catalase (CAT), superoxide dismutase (SOD), peroxidase (POD), and glutathione peroxidase (GPx) to scavenge ROS and reactive nitrogen species (RNS) in an AP rat model. In another study, Prussian blue nanozyme (PBzyme) prepared by the PVP modification method could drive intrinsic ROS scavenging and inflammation-inhibiting properties in AP therapy (Xie et al., 2021). Tang et al. (2015) synthesized Generation 5 (G5) polyamidoamine (PAMAM) dendrimers with two different surface groups, G4.5-COOH and G5-OH, and studied their therapeutic mechanisms for AP. Both dendrimers reduced pathological injuries and inflammation responses in the pancreas. A similar study was conducted by Wang et al. (2022) in which tetrahedral framework nucleic acids were developed for suppressing inflammation and preventing pathological cell death caused by AP. Based on previous studies that have reported carbon monoxide (CO)-mediated regulation of inflammatory responses and oxidative stress effectively (Otterbein et al., 2000; Zuckerbraun et al., 2007), CO-bound hemoglobin vesicles (CO-HbV) were synthesized for AP treatment (Nagao et al., 2016). CO-HbV could serve as a CO donor for driving inflammation inhibition effect and an oxygen carrier after releasing CO, exerting oxidation prevention effect. In addition, a few biomaterial-based nanoparticles were utilized only as carriers for specific drugs without studying their biodistribution to support any targeting effect (Cervin et al., 2009).

## 3 Conclusions and perspectives

AP is a life-threatening severe inflammatory disease with no clear pathogenesis. However, it is mainly characterized by inflammation infiltration and oxidative stress. Recent reports on AP treatment have primarily focused on inhibiting inflammatory responses and preventing oxidation in pancreatic tissues. With the development of nanotechnology, the biomaterials-based nanoagents may achieve passive (e.g., ELVIS and Bionic effect, pH, ROS and enzyme-dependent) or active (specific ligand or non-serum based biomarkers dependent) targeting and other functional effects (e.g., multi-enzyme activities, anti-inflammation and anti-oxidation effect) for AP treatment. Future research should be inclined towards the following aspects: (Petrov and Yadav, 2019) Studies on biomaterial-based nanoagents with active targeting to aid the release and accumulation of nanoagents in injured pancreatic tissues (Lankisch et al., 2015); Advantages of nanocarriers should be integrated for precise AP treatment to reduce side effects (Ratia Gimenez et al., 2014); Studies should be carried out in AP animal models with information on pathology and statistical data to obtain convincing results.
